# A diffuse traumatic neuroma in the palate: a case report

**DOI:** 10.1186/s13256-016-0908-5

**Published:** 2016-05-11

**Authors:** Takanori Eguchi, Rikuma Ishida, Hironori Ara, Yoshiki Hamada, Ikuyo Kanai

**Affiliations:** 1 Department of Oral and Maxillofacial Surgery, Toshiba Rinkan Hospital, 7-9-1 Kamitsuruma Minami-ku, Sagamihara, 252-0385 Japan; 2 Department of Oral and Maxillofacial Surgery, School of Dental Medicine, Tsurumi University, 2-1-3 Tsurumi Tsurumi-ku, Yokohama, 230-8501 Japan

**Keywords:** Traumatic neuroma, Amputation neuroma, Palate, Greater palatine nerve, Diffuse

## Abstract

**Background:**

A traumatic neuroma is not a true neoplasm but a reactive proliferation of neural tissue that commonly occurs after the transection or damage of a nerve bundle. Traumatic neuromas are rare in the oral region and usually occur as a solitary nodule of the mental foramen, lower lip, or tongue. This is the first report of a diffuse traumatic neuroma of the palate.

**Case presentation:**

A 30-year-old Japanese man was referred to our clinic complaining of painful swelling of the left side of his palate. The swelling was diffuse and his pain increased with palpation of his palate. He had no noteworthy medical or family history, and was not aware of any history of trauma or inflammation in his head or neck area. We administered antibiotics and non-steroidal anti-inflammatory drugs because we suspected that his symptoms were the result of inflammation caused by an infection. However, his symptoms did not change. An incisional biopsy was performed, and histopathologic examination indicated that the lesion was a traumatic neuroma. Under general anesthesia the lesion was resected with a 5-mm margin using an electric scalpel because of the diffuse expansion and indistinct borders of the mass. Some tumor cells were observed within the surgical margins of the resected specimen, but there has been no recurrence of either the pain or mass in the 3 years since the surgery.

**Conclusions:**

The location and diffuse nature of this traumatic neuroma are both very rare. While we were initially unsure about the diagnosis and treatment of this mass, the treatment outcome has been good. However, a postoperative recurrence can occur at any time following the excision of a traumatic neuroma, and close long-term follow-up will continue.

## Background

A traumatic neuroma is a hyperplastic lesion caused by trauma or surgery that involves the peripheral nerves and is not considered to be a true neoplasm [1]. It may occur in any part of the body, including the head, neck, gallbladder and thigh [2]. The clinical features of a traumatic neuroma include the formation of a solitary nodule less than 2 cm in diameter, neuralgic pain, tenderness, paresthesias and increased pain on palpation over the lesion [2, 3]. The recommended treatment of a traumatic neuroma is simple excision rather than nerve resection or alcohol blocks [4]. In the oral region, a traumatic neuroma is a rare disorder that occurs most commonly at the mental foramen, lower lip, tongue and intra-osseous areas [4, 5]. It is extremely rare in the palate. There have been no previously published reports of a diffuse traumatic neuroma. Here we describe the treatment outcomes of an unusual diffuse traumatic neuroma occurring in the palate.

## Case presentation

**Fig. 1:**
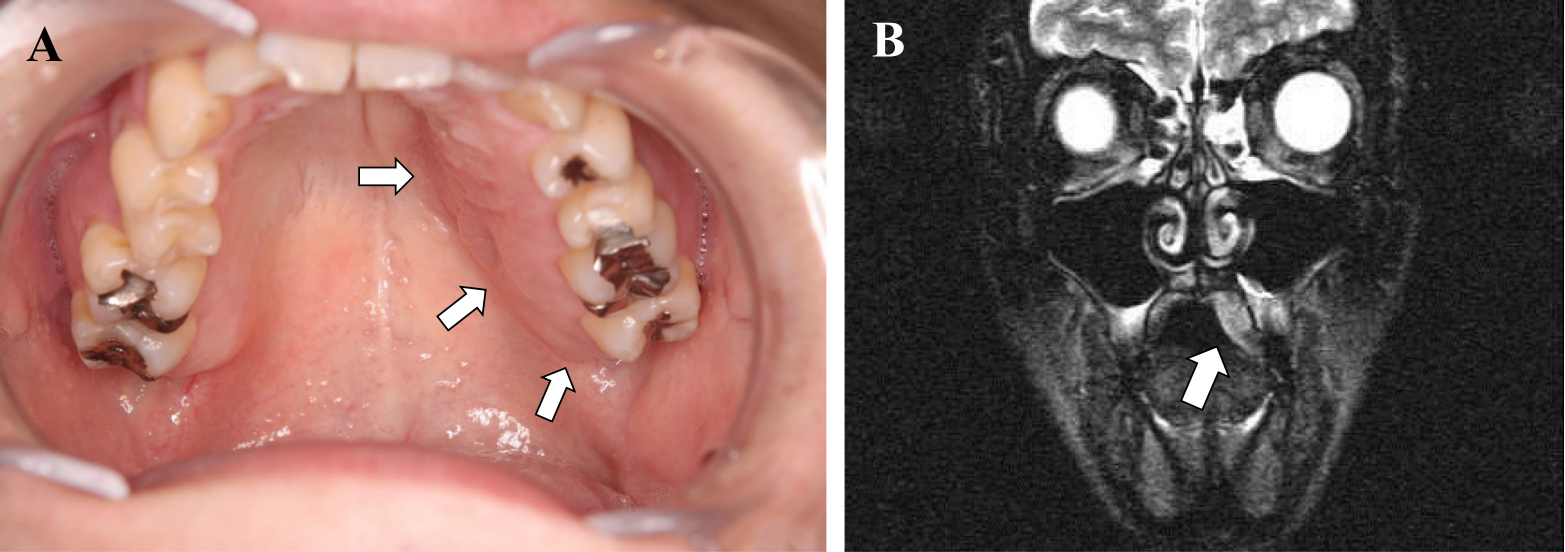
**a** Photograph showing an obvious swelling of the left palatine mucosa (*arrows*). **b** A T2-weighted magnetic resonance image showing the lesion as an area of high-signal intensity (*arrow*)

A 30-year-old Japanese man was referred to our clinic complaining of pain, tenderness, and swelling on the left side of his palate for the past several months. Our initial clinical examination found that his left palatine mucosa was significantly swollen compared with his right side. The swelling was diffuse, and its borders were unclear (Fig. 1a). The swelling was especially pronounced in the left molar region of his palate, and his pain was exacerbated with the application of direct pressure to the lesion. The swelling exhibited increased signal intensity on T2-weighted magnetic resonance images (MRI, Fig. 1b). All of his left maxillary teeth were healthy, and no specific abnormalities in his left maxilla and maxillary sinus were observed on panoramic X-ray and computed tomography (CT) images. His medical and familial histories were unremarkable, and he was not aware of any history of trauma or inflammation of his head or neck. We administered antibiotics orally (cefcapene pivoxil hydrochloride hydrate 100 mg tablet every 8 hours) and non-steroidal anti-inflammatory drugs (loxoprofen sodium hydrate 60 mg tablet every 8 hours) for 7 days because we suspected that his symptoms were due to inflammation secondary to an infection. However, his symptoms did not improve.

**Fig. 2:**
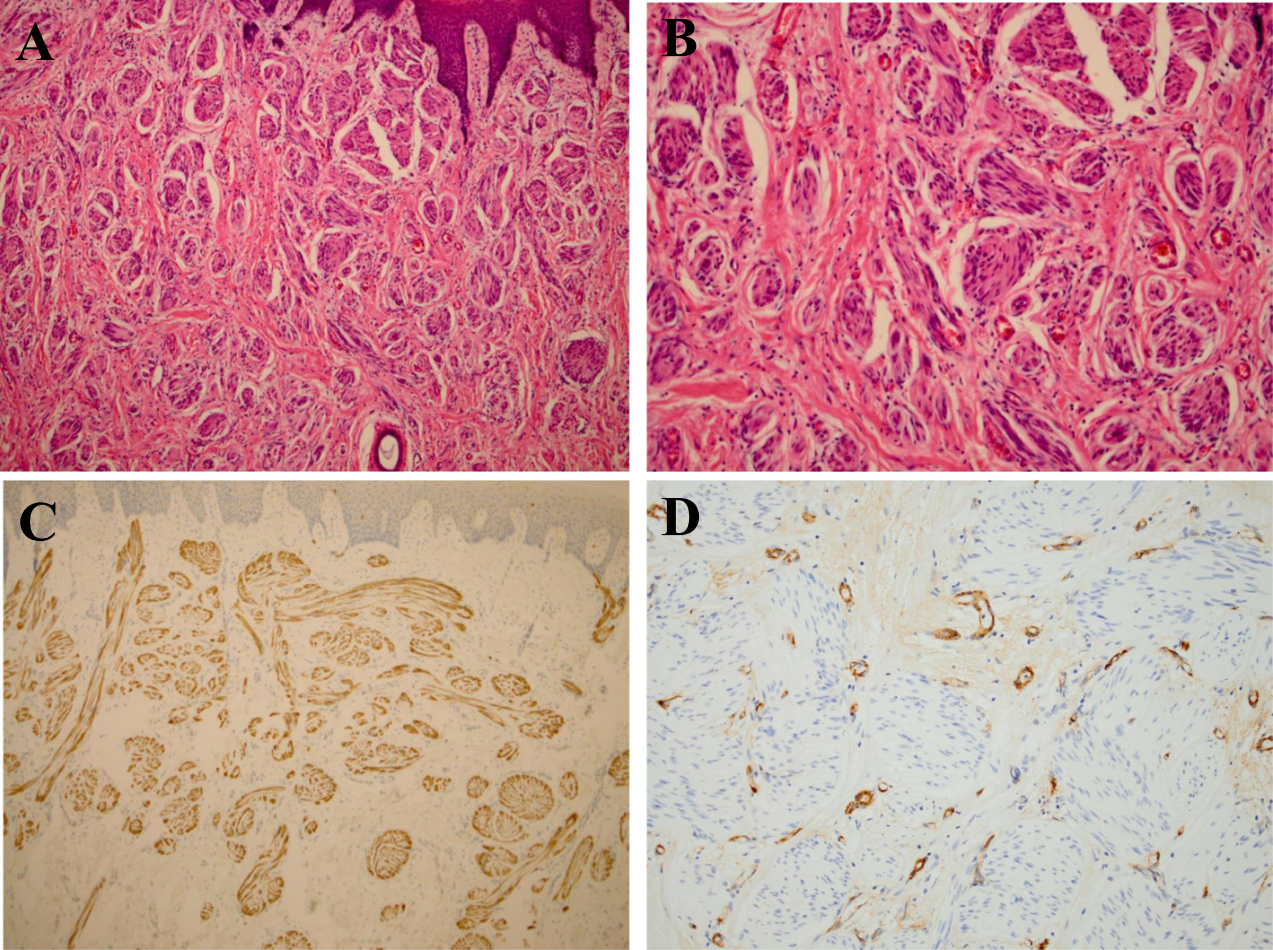
**a** Many haphazard nodes can be seen within the fibrous stroma (hematoxylin and eosin stain ×100) and **b** hematoxylin and eosin stain × 200. **c** A strong positive signal for S-100 of the bundles within the nodes is evident (S-100 protein ×100). **d** Factor VIII was positive in the microvessels within the fibrous stroma, but did not stain the nerve bundles (Factor VIII ×200)

**Fig. 3:**
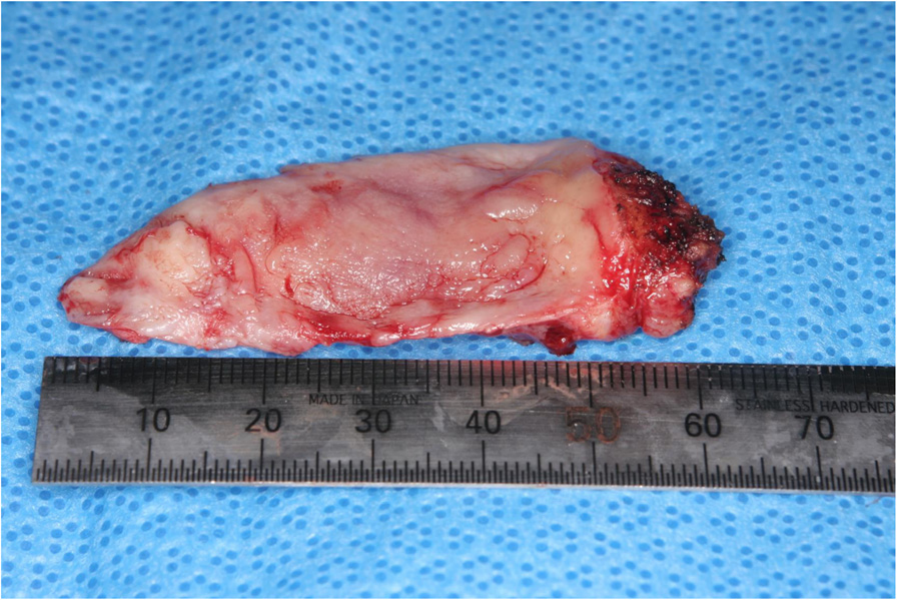
The lesion excised from the left side of the palatine mucosa was approximately 6×3 cm

Based on his clinical and radiographic examinations, our initial diagnosis was a soft tissue tumor. An incisional biopsy was performed and histopathologic examination of the lesion revealed haphazard nodes and inflammatory cells in a fibrous stroma (Fig. 2a, b). Immunohistochemical analysis revealed significant staining for the neural marker S-100 in the bundles within the node (Fig. 2c). Factor VIII staining was positive in the fibrous stroma, but not in the bundles (Fig. 2d). These findings led to the diagnosis of a traumatic neuroma. The patient underwent resection of the tumor with a 5-mm margin using an electric scalpel under general anesthesia. Although the border of the mass was unclear and diffuse, the extent of the tumor could be determined based on the MRI images. As the tumor was conglutinated with a part of his palatine bone, we saucerized the bone surface including the overlying mucosa and the periosteum. His left greater palatine nerve was resected as the possible origin of the tumor. The open surgical wound was covered with a collagen-based artificial dermis (Terudermis, Olympus Terumo Biomaterials Corp, Tokyo, Japan) and a surgical splint. The size of the specimen was approximately 6×3 cm (Fig. 3). The pathologic findings of the surgical specimen were the same as those of the incisional biopsy specimen, and the patient’s greater palatine nerve exhibited no pathologic changes. The tumor was not entirely encapsulated and some tumor cells were observed within the surgical margins. We decided to continue strict clinical follow-up without additional surgery because the traumatic neuroma is not a true neoplasm, and his pain subsided immediately after the surgery. No clinical evidence of a recurrence has been observed in the 3 years since the surgery.

## Discussion

Traumatic neuromas, also called amputation neuromas, are not true neoplasms but reactive proliferations of neural tissue that occur after transection or damage to a nerve bundle. Jones and Franklin [6] reported that the frequency of traumatic neuromas was 0.34 % in the oral region. The most common sites for a traumatic neuroma in the head and neck are the inferior alveolar nerve, lingual nerve, and great auricular nerve [2]. There have been few reports of traumatic neuromas in the palate.

The history of trauma or surgery is very important in the diagnosis of a traumatic neuroma. Our patient was not aware of any history of trauma or surgery. We therefore ruled out other neurogenic tumors (Table 1). The histologic differential diagnosis for oral traumatic neuromas includes mucosal neuromas (multiple endocrine neoplasia type 2B), neurofibromas, palisading neuromas, and neurovascular hamartomas. On histological examination, mucosal neuromas resemble traumatic neuromas because of the presence of many nerve bundles [7]. However, the absence of inflammatory cells in a normal or loose fibrous connective tissue background can lead to the diagnosis of a mucosal neuroma. Neurofibromas and traumatic neuromas both have fibrous connective tissue and non-encapsulated lesions, but neurofibromas have mast cells and nuclei with a wavy or serpiginous prolife, and do not contain the abundant, haphazardly arranged axons that are unique to traumatic neuromas [8]. Although palisading neuromas and traumatic neuromas both form nerve bundles [9], the absence of inflammatory cells and fibrous connective tissue and the existence of spindle cells showing palisading arrangement and generally circumscribed margin is more indicative of a palisading neuroma. Neurovascular hamartomas are rarely described in the oral cavity. The histopathologic features of neurovascular hamartomas include poorly circumscribed masses of closely packed nerve bundles and blood vessels in a loose matrix, containing minimal to no inflammation [10]. Given all the histologic similarities between these masses, identifying a traumatic neuroma is especially difficult. Allon *et al*. [10] noted that neurovascular hamartomas are unique histologically because their neural and vascular components are separate, with a dominant neural component. The histopathologic features of the mass described in our case were typical of a traumatic neuroma, which consists of a non-encapsulated lesion, few inflammatory cells infiltrating the stroma, and a large number of haphazardly arranged nerve fascicles within a fibrous connective tissue stroma. Immunohistochemical staining for factor VIII suggested that the neural and vascular components were separate. In addition, palpable bulging and pain on palpation are signs of a traumatic neuroma. Neurogenic tumors of the head and neck generally do not produce neurologic signs or symptoms [1, 3]. These features confirmed the diagnosis of a traumatic neuroma.

**Table 1:** Clinical and histopathological differential diagnosis of traumatic neuroma versus other neurogenic tumors

	Clinical features	Histopathological features
Traumatic neuroma	Symptomatic (anesthesia, dysesthesia, and pain), solitary	Many nerve bundles, fibrous connective tissue background containing inflammatory cells
Mucosal neuroma	Asymptomatic, typically multiple, associated with multiple endocrine neoplasia type 2B	Many nerve bundles, normal or loose fibrous connective tissue background without inflammatory cells
Neurofibroma	Asymptomatic, solitary, or multiple	Nuclei with wavy or serpiginous prolife, fibrous connective tissue background containing mast cells
Palisading neuroma	Asymptomatic, solitary	Circumscribe, spindle cells showing palisading arrangement
Neurovascular hamartoma	Asymptomatic, solitary	Many nerve bundles containing vessels, fibrous connective tissue background without inflammatory cells

The palatine mucosa is composed of a keratinized stratified squamous epithelium, lamina propria, and a submucosal layer. The submucosal layer is composed of glandular and adipose tissues that surround the palatine neurovascular bundle that runs under the lamina propria [11]. The palatine mucosa is innervated by the nasopalatine nerve and the greater palatine nerve. The nasopalatine nerve is a branch of the sphenopalatine nerve, and innervates the palatal tissues and gingiva anterior to the canines after passing through the incisive canal [12]. The greater palatine nerve is the anterior branch of the palatine nerve, and innervates the palatal tissues and gingiva posterior to the canines after passing through the greater palatine foramen [13]. Both nerves are sensory nerves, and a traumatic neuroma commonly occurs in sensory nerves [2]. In our patient, a traumatic neuroma developed in the left side of the palatine mucosa of his molar region, and his pain was limited to his posterior palatal gingiva. His greater palatine nerve was not cut, and histopathologic examination revealed that the trunk of his greater palatine nerve had no pathologic changes. Based on those findings, the origin of his neuroma was thought to be a branch of his left greater palatine nerve.

Causal factors for oral traumatic neuromas include tooth extraction, orthognathic surgery, ill-fitting dentures and intra-oral incisions [1, 3, 14]. In our case, however, there was no history of trauma or surgery according to the patient. However, the palatine mucosa is easily injured by burns and mechanical trauma during eating, resulting in a compounded thermal and mechanical trauma [15]. As this patient’s traumatic neuroma was diffusely expanded within the palate, it may have been induced by a broader nerve injury such as a burn.

It is a possibility that our surgical treatment did not result in the complete excision of the lesion. However, the tumor has not recurred in the 3 years since the surgery. Tay *et al*. [16] reported that monopolar diathermy reduces the rate of neuroma formation, and electrical coagulation of the proximal nerve stump can prevent the development of neuromas [16]. Simple excision is therefore highly recommended in the treatment of traumatic neuromas [1].

The borders of diffuse traumatic neuromas are often unclear, and the extent of the actual tumor may be such that a complete excision would result in severe neurologic damage, such as an area of hypoesthesia or complete nerve palsy. In these cases, simple excision of the tumor using an electrical scalpel is an effective method of treatment, and reduces the likelihood that the residual traumatic neuroma tissue would cause repeat symptoms or a full recurrence of the tumor.

## Conclusions

We report an extremely rare traumatic neuroma in an uncommon location and without a defined cause.

## Consent

Written informed consent was obtained from the patient for the publication of this case report and any accompanying images. A copy of the written consent is available for review by the Editor-in-Chief of this journal.
